# Overground Robot-Assisted Gait Training for Pediatric Cerebral Palsy

**DOI:** 10.3390/s21062087

**Published:** 2021-03-16

**Authors:** Seung Ki Kim, Dongho Park, Beomki Yoo, Dain Shim, Joong-On Choi, Tae Young Choi, Eun Sook Park

**Affiliations:** 1 Department and Rehabilitation Medicine, Yongin Severance Hospital, Yonsei University College of Medicine, 363 Dongbaekjukjeon-daero, Giheung-gu, Yongin-si 16995, Korea; gsg1230@yuhs.ac; 2 Department and Research Institute of Rehabilitation Medicine, Severance Hospital, Yonsei University College of Medicine, 50-1 Yonsei-ro, Seodaemun-gu, Seoul 03722, Korea; dhparkmed@yonsei.ac.kr (D.P.); beomkey@yuhs.ac (B.Y.); sdi3807@yuhs.ac (D.S.); supermanjo12@yuhs.ac (J.-O.C.); PIYO1214@yuhs.ac (T.Y.C.)

**Keywords:** exoskeleton, robotic training, gait, cerebral palsy, pediatric

## Abstract

The untethered exoskeletal robot provides patients with the freest and realistic walking experience by assisting them based on their intended movement. However, few previous studies have reported the effect of robot-assisted gait training (RAGT) using wearable exoskeleton in children with cerebral palsy (CP). This pilot study evaluated the effect of overground RAGT using an untethered torque-assisted exoskeletal wearable robot for children with CP. Three children with bilateral spastic CP were recruited. The robot generates assistive torques according to gait phases automatically detected by force sensors: flexion torque during the swing phase and extension torque during the stance phase at hip and knee joints. The overground RAGT was conducted for 17~20 sessions (60 min per session) in each child. The evaluation was performed without wearing a robot before and after the training to measure (1) the motor functions using the gross motor function measure and the pediatric balance scale and (2) the gait performance using instrumented gait analysis, the 6-min walk test, and oxygen consumption measurement. All three participants showed improvement in gross motor function measure after training. Spatiotemporal parameters of gait analysis improved in participant P1 (9-year-old girl, GMFCS II) and participant P2 (13-year-old boy, GMFCS III). In addition, they walked faster and farther with lower oxygen consumption during the 6-min walk test after the training. Although participant P3 (16-year-old girl, GMFCS IV) needed the continuous help of a therapist for stepping at baseline, she was able to walk with the platform walker independently after the training. Overground RAGT using a torque-assisted exoskeletal wearable robot seems to be promising for improving gross motor function, walking speed, gait endurance, and gait efficiency in children with CP. In addition, it was safe and feasible even for children with severe motor impairment (GMFCS IV).

## 1. Introduction

Gait impairment is an important treatment target in children with cerebral palsy (CP). Robot-assisted gait training (RAGT) has been used over the past decade to help improve gait function [1,2]. This training provides conditions that support motor learning principles (such as intensity, repetition, task specificity, and participation) to promote both neuroplastic changes and nonmotor recovery in patients with central nervous system–related gait disorders. To train gait in children with CP, tethered exoskeletal systems, such as Lokomat (Hocoma AG, Volketswil, Switzerland), have been used [1]. This device exerts force through a rigid articulated frame that moves the patient’s leg in one or more planes with a weight support system [3]. However, there is still insufficient evidence that RAGT provides better treatment outcomes for gross motor function and walking abilities than conventional physical therapy [1,2]. Because it only moves the patient according to a predetermined fixed trajectory or timing, the tethered exoskeleton can restrict the patient’s active participant to make the passive, thereby reducing muscle activity [4]. In addition, patterns of abnormal muscle activity may appear when the patient actively walks and resists the device [5].

By contrast, untethered exoskeletons, such as ReWalk (ReWalk Robotics Inc., Marlborough, MA, USA), Indego (Parker Hannefin Corp, Mayfield Heights, OH, USA), Hybrid Assistive Limb (HAL; Cyberdyne Inc, Tsukuba, Japan), and the Ekso (Ekso Bionics, Richmond, CA, USA), are wearable, articulated suits that transmit power and their own power source and control algorithms, providing patients with the most freedom and realistic walking experience [3]. These devices are used for gait assistance and rehabilitation, and several studies have shown the effect of gait training in patients with gait impairment due to various central nervous system diseases [6].

Untethered exoskeletal robot could provide task-specific overground training. Overground gait training using an untethered exoskeleton may encourage active patient participation proved to be exciting and challenging [7]. Small-sized studies for patients with stroke or spinal cord injury reported that walking speed and distance improved after RAGT [8,9,10]. After gait training using HAL, stroke patients showed improvement in the 10 m walk test and functional ambulation categories [10], and other studies also suggested that walking ability and trunk posture could be improved [8]. Overground RAGT using the Ekso showed improved walking speeds and distances in three patients with chronic and complete spinal cord injury [9]. A case study with an 11-years-old female with incomplete paraplegia reported that she increased walking speed, decreased oxygen consumption, and improved the range of motion of the hip and knee joints during walking with the robot after gait training using Angel-suit (ANGEL ROBOTICS Co., Ltd., Seoul, Korea) [11]. Several studies reported early evidence that walking with an untethered exoskeleton reduces immobility, improves spasticity and cardiopulmonary function, and has a positive effect on the musculoskeletal system [8,9,12,13,14].

However, few studies have examined overground RAGT using untethered exoskeletons in children with CP [15,16,17]. Gait training using HAL, which assists torque in hip and knee joints, showed improvement of gross motor function and walking ability in six adolescents with CP [15,17]. Robotic gait training with the exoskeleton assisting knee extension showed no significant changes in gait speed and knee extension in null exoskeleton condition in seven children with crouch gait from CP [16]. Therefore, this pilot study examined overground RAGT using a torque-assisted exoskeletal wearable robot in children with CP and evaluated its safety and effectiveness.

## 2. Materials and Methods

### 2.1. Wearable Torque-Assisted Exoskeletal Robot

**Figure 1:**
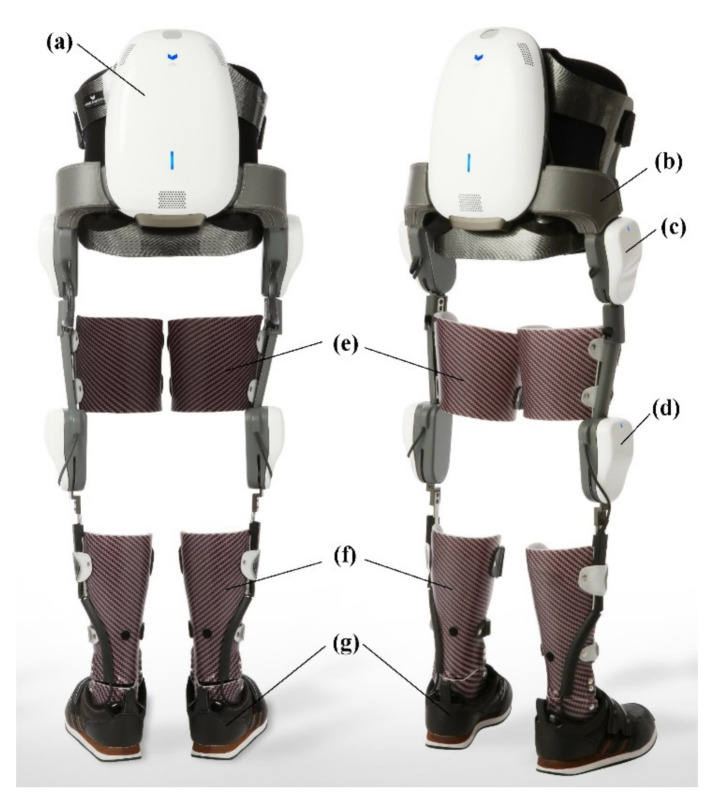
The powered exoskeleton used in the clinical experiments. (**a**) A controller back-pack including a battery, (**b**) a pelvic frame that rigidly supports the backpack and connects two hip joint actuators, (**c**) hip joint actuators, (**d**) knee joint actuators, (**e**) thigh bands, (**f**) ankle-foot-orthosis, and (**g**) shoes. The ground contact sensors are installed under the sole of the ankle-foot-orthosis.

This study used Angel-legs (ANGEL ROBOTICS Co., Ltd., Seoul, Korea), a wearable torque-assisting exoskeleton robot which was a gait training version of Angel-suit designed to assist walking in patients with incomplete paraplegia [11]. This device can generate assistive torques according to gait phases automatically detected by force sensors installed beneath the ankle-foot-orthosis (Figure 1f,g). The joint actuators of Angel-legs, shown as in Figure 1c,d, generate flexion torque during the swing phase and extension torque during the stance phase, respectively, at both the hip and knee joints. The remaining ankle joint is mechanically controlled using the articulated ankle-foot-orthosis (Figure 1f).

The robot provides a transparent assistance by utilizing a low inertia motor and an impedance reduction control algorithm. This makes it possible to minimize discomfort by preventing unexpected resistance to the spontaneous movement of a patient with incomplete paraplegia. The actuator modules consist of brushless direct-current motors (70 W, EC45-flat, Maxon motor Ltd., Sachseln, Switzerland), customized gear sets, and sensors for measuring the motor angular position and the absolute angle of the human joint. The magnitude of an assistive torque is approximately 17 Nm in a continuous assistance condition.

### 2.2. Participants

Children with spastic CP were recruited according to the study inclusion and exclusion criteria. The inclusion criteria were patients with spastic CP aged between 7 and 18 years and diagnosed as having gait abnormality with lower limb weakness. The exclusion criteria were as follows: (1) inability to understand and follow commands, (2) spasticity of the lower extremities greater than 3 (as evaluated on the modified Ashworth scale), and (3) any contracture, deformity, or skin problem in the lower extremities that would affect wearing and walking with the robot.

Ethical approval was granted by the Institutional Review Board and Ethics Committee of the Severance Hospital (#4-2016-0167). Informed consent was obtained from the primary caregiver and/or the participant along with written minor assent according to the rules of the IRB of our hospital.

### 2.3. Training Protocol

Overground RAGT was conducted for 17~20 sessions (60 min per session, 1~3 sessions per week) in each child. First, children practiced sit-to-stand movement and walking with parallel bars for additional assistance and stability to adapt to the robot. Next, walking training was performed using a wheeled walker or bilateral crutch. One child used a walker with a harness or platform walker during training and evaluation because she was unable to walk without these walking devices. All training was conducted by a physical therapist with over 10 years of experience in CP treatment. During the training period, the participants were required to maintain the same amount and level of the physical therapy as before.

### 2.4. Clinical Evaluation

A clinical evaluation was performed in participants without the robot before and after the training to measure (1) the motor functions using the gross motor function measure (GMFM) and the pediatric balance scale, and (2) the gait performance using instrumented gait analysis, the 6-min walk test (6MWT), and oxygen consumption. In addition, the clinical evaluation included each child’s height, weight, range of motion, muscle strength, and spasticity.

#### 2.4.1. Gross Motor Function Measure

The GMFM is a standardized observational assessment tool with validity that measures changes in gross motor function over time in children with CP. It is a four-point scale divided into five dimensions (lying and rolling; sitting; crawling and kneeling; standing; and walking, running, and jumping). The sum of items of each dimension was indicated as a percentage [18].

#### 2.4.2. Pediatric Balance Scale

The pediatric balance scale is a modified version of the Berg balance scale for evaluating functional balance skills in children. The scale consists of 14 items that are scored from 0 points (lowest function) to 4 points (highest function) with a maximum score of 56 points [19]. Pediatric balance scale (PBS) was evaluated only for subjects who could stand alone for a while.

#### 2.4.3. Spatiotemporal Parameters of Gait

Spatiotemporal parameters of gait were measured using a computerized motion analysis system (VICON MX-T10 Motion Analysis System, Oxford Metrics Inc., Oxford, UK). Using the Helen Hayes marker set, 16 passive reflex markers were placed on each participant by a trained investigator with 20 years of clinical experience in gait analysis. While the child walked on an 8-m pathway, six digital videos on the front, rear, and sides were recorded simultaneously. Each child was instructed to look straight ahead and walk as naturally as possible. Data were collected at a sampling rate of 100 Hz from three trials at self-selected walking speed. The parameters included cadence, walking speed, step length, step time, step width, stride length, stride time, and single and double limb support durations. Then, the mean values were calculated from the three repetitions for each child. Each participant who needed a walking device at the initial evaluation used the same assistive device at all assessment sessions.

#### 2.4.4. 6-Min Walk Test

Gait endurance was assessed using the 6MWT [20]. The 6MWT was reliable and valid for children with CP [20,21,22]. Participants walked for 6 min at their self-selected speed and could rest when they felt unable to continue. The total walking distance was recorded. Use of any physical assistive device was documented. Each participant used the same assistive device at all assessment sessions. The distance covered during the 6MWT was measured in meters.

#### 2.4.5. Oxygen Consumption

Oxygen consumption and heart rate data were acquired and recorded using a Cosmed K5 metabolic monitoring unit (Cosmed, Italy). Oxygen consumption had satisfactory reliability in patients with CP [23,24,25]. The Cosmed K5 is a lightweight, portable unit that records heart rate from a Polar chest belt monitor and samples oxygen and carbon dioxide concentrations during inspiration and expiration through a facemask. Prior to the exercise test, the K5 mask was placed over the participant’s mouth and nose. No warm-up regimen was performed by the participant. The baseline oxygen consumption (measured as VO2/min) and heart rate were recorded for 5 min, while the participant was seated. Next, oxygen consumption was measured during the 6MWT. Oxygen consumption measurements were averaged over the entire 6-min period rather than only when consumption reached steady state at the end of the period. Oxygen rate, measured as volume of oxygen consumed per unit of body weight in 1 min (mL/kg/min), provides an index of intensity of physical work at any given time. Oxygen cost was determined by dividing the rate of oxygen consumption by the speed of walking. Oxygen cost is a precise indicator of efficiency of gait, the amount of energy expended to walk over a standard distance (mL/kg/m) [26]. The 6MWT and oxygen consumption were evaluated at baseline, after eight sessions, and at the end of the training.

## 3. Results

A total of three participants diagnosed with bilateral spastic CP participated in this study: P1, a 9-year old girl with Gross Motor Function Classification System (GMFCS) level II, P2, a 13-year old boy with GMFCS level III, and P3, a 16-year old girl with GMFCS level IV. P3 had mild mental retardation, but there were no problems with training. All participants did not have any other visual, neurological (e.g., seizure) or musculoskeletal problems that could affect RAGT. They were undergoing conventional rehabilitation therapy for more than 8 to 15 years after diagnosis of bilateral spastic CP. They maintained physical therapy twice a week (60 min per session) during the study period from at least 3 months before participation in this study, and occupational therapy once a week (30 min per session) was additionally maintained for P2 and P3. P1 received 20 RAGT sessions in 20 weeks; P2, 17 RAGT sessions in 12 weeks; and P3, 18 RAGT sessions in 7 weeks (Table 1). No safety issues were noted during the training period, and participants did not complain of any important side effects.

### 3.1. Gross Motor Function & Balance

All participants showed improvement in gross motor function. In the GMFM, participant P1 showed improvement from 58.97% to 71.79% in dimension D (standing) and from 78.9% to 81.46% in the total average; no change was observed in dimension E (walking, running, and jumping). Participant P2 exhibited improvement from 96.8% to 100% in dimension A (lying and rolling), from 29.17% to 31.94% in dimension E, and from 73.99% to 75.33% in the total average; no change was found in dimension D. Participant P3 demonstrated improvement from 40.48% to 47.62% in dimension C (crawling and kneeling), from 0% to 2.56% in dimension D, and from 40.53% to 42.47% in the total average; no change was seen in dimension E. Minimal clinically important differences (MCIDs) were reported for dimensions D and E only in patients with CP [27]. According to these results, participants P1 and P3 showed MCIDs in dimension D, and participant P2 exhibited a MCID in dimension E (Table 2).

Participants P1 and P2 exhibited improvement in the pediatric balance scale evaluation after treatment. Participant P1 improved from 19 to 22 in the dynamic score and from 40 to 43 in the total score. Participant P2 improved from 14 to 15 in the static score, from 13 to 14 in the dynamic score, and from 27 to 29 in the total score. Participant P3 could not be evaluated before and after treatment for this scale (Table 2).

### 3.2. Spatiotemporal Parameters of Gait

Gait analysis was only available in participants P1 and P2 because participant P3 was not able to walk. For participant P1, cadence increased by 15.5%, speed increased by 3.6%, and stride length decreased by 11.0%. For participant P2, cadence increased by 311.9%, speed increased by 442.9%, and stride length increased by 30.8% (Table 3). Participant P2 reached a MCID in cadence, speed velocity, and stride length, and participant P1 reached a MCID in cadence [27,28].

### 3.3. Gait Endurance and Oxygen Consumption

**Figure 2:**
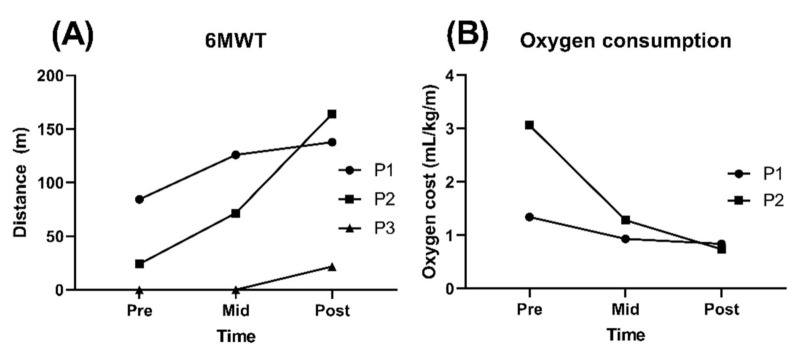
Changes in the 6-min walking test (6MWT) (**A**) and oxygen consumption (**B**) in children with CP through before, after 8 sessions, and at the end of robot-assisted gait training (RAGT). In the 6MWT, participant P1 walked with no device, participant P2 walked with bilateral crutches, and participant P3 used a platform walker. The oxygen cost was measured during the 6MWT. Participant P3 did not perform the oxygen consumption test due to complaints of discomfort with the mask while walking.

All participants showed an increase in walking distance during the 6MWT after RAGT. Participant P1 increased by 53.48 m (63.4%), and participant P2 increased by 140.16 m (580.6%). Participant P3 needed the continuous help from a therapist for stepping at baseline even when wearing the harness or using a platform walker, but she was able to walk with platform walker independently. Participant P3 improved to a walking distance of 45.2 m with the harness and 21.78 m with a platform walker during the 6MWT (Table 4 and Figure 2A).

The oxygen cost was evaluated during the 6MWT for participants P1 and P2. In participant P3, the test was not measured due to complaints of discomfort with the mask while walking. Participant P1 decreased oxygen cost by 37.3% (from 1.34 to 0.84), and P2 decreased this measure by 75.8% (from 3.06 to 0.74; Table 4 and Figure 2B). The MCID (large effect size) for oxygen cost was 0.17 in GMFCS level II patients and 0.09 in GMFCS level III patients, and both participants P1 and P2 reached a MCID.

## 4. Discussion

This study examined changes in gross motor function and walking ability after RAGT using a torque-assisted wearable exoskeletal robot in three children with different GMFCS levels. After this training, all participants showed great improvement in gross motor function and walking ability. In addition, during this training period, none of the participants complained of side effects, such as skin abrasion or discomfort, while using the exoskeletal robot.

Most studies on RAGT in children with CP have used robots with a tethered exoskeleton [1,2]. These robots are similar to traditional body weight-supported treadmill training but maintain the proper alignment and pattern when the patient walks by giving specific guidance to hip, knee, or ankle movements using an exoskeleton or footplate [29]. Several studies have reported that RAGT using robots with a tethered exoskeleton show improvements in gait speed, gait endurance, and gross motor function in individuals with CP. However, these results were not considered statistically significant in a meta-analysis [1]. A systematic review found weak and inconsistent evidence that RAGT is a benefit in children with gait disorders, especially CP, and did not show superior effects compared to conventional physiotherapy [2]. In other study, RAGT did not show improvement in standing ability or gait function in patients with CP of GMFCS level III–IV [30]. Willoughby et al. reported that the weight support by the harness and the promotion of the step by the movement of the treadmill belt might make training less challenging and less intense than overground walking. In addition, the improvement effect of gait function could not be directly transmitted to overground walking because treadmill training does not provide task-specific training for overground walking environment [31].

The untethered exoskeleton enables active overground walking in response to the wearer’s intention. The Angel-legs used in this study has actuators in the hip and knee joints, which assist the joint torque generation according to the gait cycle of the wearer by force sensor pad. The HAL assists the hip and knee joints based on patient’s voluntary muscle activities (hip and knee flexors and extensors) via electromyography electrodes. Studying the effect of gait training using HAL in six adolescents with CP of GMFCS level II~IV, Matsuda et al. reported that increased GMFM, walking speed, cadence, single limb support, 6-min walking distance, and hip joint angle in the swing phase [15]. Ueno et al. reported that eight adolescents and adults with bilateral spastic CP of GMFCS level III~IV showed increases in comfortable gait speed, step length, and cadence after 8 sessions of overground RAGT using HAL [32]. In a case study using HAL, 12 sessions of overground RAGT improved walking ability, gross motor function in an 11-year-old boy with GMFCS level IV, and the effects were maintained for at least 3 months after training [17]. Like the studies using HAL, overground RAGT using Angel-legs increased in GMFM, gait speed, cadence, and 6-min walking distance. In this study, the step length of the patient with GMFCS level II decreased, and the step length of the patient with GMFCS level III increased. Studies using HAL did not show any significant changes in a study on patients with GMFCS level II~IV [15], and in a study on patients with GMFCS level III~IV, there was a significant increase [32]. Lerner et al. evaluated the effect of the robotic exoskeleton assisting knee extension during overground walking in seven children with crouch gait from CP of GMFCS level I~II. They reported improved knee extension and increased step length and gait speed without decreased volitional muscle activity when wearing the exoskeleton. In addition, despite the short training period of two 2–3-h training sessions, the children exhibited significantly increased step length and knee extension at initial contact even when not wearing the robot [16]. However, it is difficult to directly compare with this study because the training period is short to find out the effect of overground RAGT.

In this study, the participants not only increased gait speed but also increased gait endurance and reduced energy expenditure. The changes were smaller in the child with GMFCS level II (P1) than in the child with GMFCS level III (P2). This result differed from previous studies, which had conflicting results on changes by level. Borggraefe et al. reported that CP patients with GMFCS level III or IV showed less improvement in dimension E of the GMFM than those with GMFCS level I or II after RAGT using a tethered exoskeleton [30]. In the study of RAGT using HAL which was an untethered exoskeleton, the change in walking distance was greater in mildly affected patients [15]. In addition, other studies have reported that GMFCS level III and IV patients have lower potential for gain in motor function from this treatment [33,34]. Unlike participant P2 who showed continuous improvement throughout the training period, participant P1 showed a rapid improvement in the early part of the training period in the 6MWT and oxygen cost but only a slight improvement thereafter. Moreover, participant P1 did not change in dimension E after the training. Nevertheless, improvements in balance, standing ability, and gait endurance were observed in participant P1. Additional studies should evaluate the effect of overground RAGT with an untethered exoskeleton in children with CP by GMFCS level.

The GMFCS III child (P2) who had previously been crutch walking showed steady improvement in walking distance and oxygen cost during the training. Meanwhile, the child with GMFCS IV was previously unable to walk independently, relying on the continuous assistance of the therapist even with a platform walker or a harness before treatment; however, after treatment, she was able to walk independently using these assistive devices. This suggested that the overground RAGT using an untethered exoskeleton can yield sufficient effects even for children with CP who have difficulty in independent walking and a low level of motor function.

In general, it is known that patients with CP after 7 years of age have reached a plateau in gross motor function [33]. However, this study showed improvement in gross motor function after overground RAGT in all participants aged 9–16 years. This was also shown in a study using HAL, which showed improvement in gross motor function, as well as walking ability, in patients aged 13–24 years (mean 16.8 years old) [15].

In motor learning-based RAGT methods, an important factor for performance improvements is a voluntary effort to obtain the desired behavior [35]. The untethered exoskeleton can assist voluntary walking according to the intention of the wearer to walk and can help the wearer to actively control balance, move weight, and activate muscles. Furthermore, it is thought that the training effect might carry over to actual walking by providing the same daily walking environment of overground gait training.

Children with CP require high energy expenditure during walking [36,37,38]. Cardiopulmonary training in children with CP is also important to improve motor function [39,40]. The 6MWT is a measurement of overall functional status related to the integrated response of multiple body systems, such as cardiovascular, respiratory, nervous, and muscular systems, during walking [41]. In this study, walking distance during the 6MWT increased, suggesting that cardiopulmonary and muscular endurance can improve through overground RAGT. In addition, it was suggested that gait training at the aerobic threshold shows improvement in mobility [42]. In this study, participants reported that they were out of breath and sweating during RAGT, which they had not experienced in any previous conventional physical therapy.

A previous study reported the strengthening of lower extremity muscles after RAGT [43]. Although lower extremity muscle strength was not measured in this study, participants reported mild muscle soreness in the lower extremity after training, suggesting that untethered overground RAGT enabled active use of lower extremity muscles. Therefore, enhanced muscle strength and anaerobic capacity by overground RAGT may also contribute to the improvement of gait ability and motor function.

The limitations of this study were that the lack of a control group, the variation in training period length by participants, and the small sample size of one participant per GMFCS level. This study was a pilot study to confirm the safety and effectiveness of overground RAGT using a torque-assisted exoskeletal wearable robot, so the number of participants was small and the control group was not recruited. However, considering that all of the children of this study have received rehabilitation treatment since birth, this study suggested that overground RAGT using an untethered exoskeleton had made significant changes. In the future, a randomized controlled study with larger sample size will be needed to verify these findings.

## 5. Conclusions

This pilot study showed overground RAGT using a torque-assisted exoskeletal wearable robot is a promising treatment for improving gross motor function (especially standing and walking), walking speed, gait endurance, and gait efficiency. In addition, this treatment was safe and feasible even for children with severe motor impairment (GMFCS IV). This study suggests that overground RAGT using a torque-assisted exoskeletal wearable robot may be an effective treatment for improving motor function in adolescents with CP. Further research in the form of a large randomized controlled study is required to verify these findings.

## Figures and Tables

**Table 1:** Demographic data of the participants.

Participant	Gender/ Age (Year)	Height (cm) Weight (kg)	GMFCS	Walking Aid	Training Sessions	Duration (Weeks)
P1	F/9	137/37	II	None	20	20
P2	M/13	143/53	III	Bilateral crutches	17	12
P3	F/16	154/51	IV	Harness or platform walker	18	7

Bwt, Birth weight; GMFCS, gross motor function classification system.

**Table 2:** Scores of gross motor function measure (GMFM-88) and pediatric balance scale (PBS) in children with cerebral palsy before and after robot-assisted gait training.

		GMFM-88 ^a^ (%)						PBS		
		A	B	C	D	E	Total	Static	Dynamic	Total
P1	Pre	100	100	95.24	58.97	40.28	78.90	21	19	40
	Post	100	100	95.24	71.79	40.28	81.46	21	22	43
P2	Pre	96.8	100	85.71	58.97	29.17	73.99	14	13	27
	Post	100	100	85.71	58.97	31.94	75.33	15	14	29
P3	Pre	92.16	70	40.48	0	0	40.53	0	0	0
	Post	92.16	70	47.62	2.56	0	42.47	0	0	0

^a^ The GMFM divided into five dimension: A, lying and rolling; B, sitting; C, crawling and kneeling; D, standing; and E, walking, running, and jumping.

**Table 3:** Gait spatial and temporal parameters in children with cerebral palsy (CP) before and after robot-assisted gait training.

		Assist Device	Cadence (Steps/min)	Speed (m/s)	Step Length (m)	Step Time (s)	Step Width (m)	Stride Length (m)	Stride Time (s)	SLS (% of GC)	DLS (% of GC)
P1	Pre	None	80.5	0.55	0.52	0.72	0.20	0.82	1.49	32.9	42.9
	Post		93.0	0.57	0.41	0.69	0.22	0.73	1.29	24.8	46.5
P2	Pre	Bilateral crutches	21.9	0.07	0.21	2.38	0.14	0.39	5.48	6.9	86.9
	Post		90.2	0.38	0.28	0.83	0.19	0.51	1.33	18.8	55.7

Gait analysis was only available in participants P1 and P2. DLS, double limb support; GC, gait cycle; SLS, single limb support.

**Table 4:** Gait endurance and oxygen consumption in children with cerebral palsy before and after robot-assisted gait training.

		6-Min Walking Test (m)	Oxygen Cost (mL/kg/m)
P1	Pre	84.34	1.34
	Mid	125.84	0.93
	Post	137.82	0.84
P2	Pre	24.14	3.06
	Mid	71.54	1.28
	Post	164.30	0.74
P3 ^a^	Pre	Uncheckable	Not tested
	Mid	Uncheckable	Not tested
	Post	45.20 (harness)/21.78 (platform walker)	Not tested

^a^ Oxygen consumption was not measured due to complaints of discomfort with the mask while walking.

## Data Availability

The data presented in this study are available on request from the corresponding author. The data are not publicly available due to reasons concerning privacy of the subjects.

## References

[1] Carvalho I., Pinto S.M., Chagas D.D.V., Praxedes Dos Santos J.L., de Sousa Oliveira T., Batista L.A. (2017). Robotic Gait Training for Individuals with Cerebral Palsy: A Systematic Review and Meta-Analysis. Arch. Phys. Med. Rehabil..

[2] Lefmann S., Russo R., Hillier S. (2017). The effectiveness of robotic-assisted gait training for paediatric gait disorders: Systematic review. J. Neuroeng. Rehabil..

[3] Esquenazi A., Talaty M. (2019). Robotics for Lower Limb Rehabilitation. Phys. Med. Rehabil. Clin. N. Am..

[4] Israel J.F., Campbell D.D., Kahn J.H., Hornby T.G. (2006). Metabolic costs and muscle activity patterns during robotic- and therapist-assisted treadmill walking in individuals with incomplete spinal cord injury. Phys. Ther..

[5] Hidler J.M., Wall A.E. (2005). Alterations in muscle activation patterns during robotic-assisted walking. Clin. Biomech..

[6] Esquenazi A., Talaty M., Jayaraman A. (2017). Powered Exoskeletons for Walking Assistance in Persons with Central Nervous System Injuries: A Narrative Review. PMR.

[7] Bortole M., Venkatakrishnan A., Zhu F., Moreno J.C., Francisco G.E., Pons J.L., Contreras-Vidal J.L. (2015). The H2 robotic exoskeleton for gait rehabilitation after stroke: Early findings from a clinical study. J. Neuroeng. Rehabil..

[8] Wall A., Borg J., Palmcrantz S. (2015). Clinical application of the Hybrid Assistive Limb (HAL) for gait training—A systematic review. Front. Syst. Neurosci..

[9] Kressler J., Thomas C.K., Field-Fote E.C., Sanchez J., Widerstrom-Noga E., Cilien D.C., Gant K., Ginnety K., Gonzalez H., Martinez A. (2014). Understanding therapeutic benefits of overground bionic ambulation: Exploratory case series in persons with chronic, complete spinal cord injury. Arch. Phys. Med. Rehabil..

[10] Nilsson A., Vreede K.S., Haglund V., Kawamoto H., Sankai Y., Borg J. (2014). Gait training early after stroke with a new exoskeleton--the hybrid assistive limb: A study of safety and feasibility. J. Neuroeng. Rehabil..

[11] Choi H., Na B., Kim S., Lee J., Kim H., Kim D., Cho D., Kim J., Shin S., Rha D.-W. Angel-suit: A Modularized Lower-limb Wearable Robot for Assistance of People with Partially Impaired Walking Ability. Proceedings of the 2019 Wearable Robotics Association Conference (WearRAcon).

[12] Aach M., Cruciger O., Sczesny-Kaiser M., Hoffken O., Meindl R., Tegenthoff M., Schwenkreis P., Sankai Y., Schildhauer T.A. (2014). Voluntary driven exoskeleton as a new tool for rehabilitation in chronic spinal cord injury: A pilot study. Spine J..

[13] Esquenazi A., Talaty M., Packel A., Saulino M. (2012). The ReWalk powered exoskeleton to restore ambulatory function to individuals with thoracic-level motor-complete spinal cord injury. Am. J. Phys. Med. Rehabil..

[14] Wu C.H., Mao H.F., Hu J.S., Wang T.Y., Tsai Y.J., Hsu W.L. (2018). The effects of gait training using powered lower limb exoskeleton robot on individuals with complete spinal cord injury. J. Neuroeng. Rehabil..

[15] Matsuda M., Iwasaki N., Mataki Y., Mutsuzaki H., Yoshikawa K., Takahashi K., Enomoto K., Sano K., Kubota A., Nakayama T. (2018). Robot-assisted training using Hybrid Assistive Limb(R) for cerebral palsy. Brain Dev..

[16] Lerner Z.F., Damiano D.L., Bulea T.C. (2017). A lower-extremity exoskeleton improves knee extension in children with crouch gait from cerebral palsy. Sci. Transl. Med..

[17] Kuroda M., Nakagawa S., Mutsuzaki H., Mataki Y., Yoshikawa K., Takahashi K., Nakayama T., Iwasaki N. (2020). Robot-assisted gait training using a very small-sized Hybrid Assistive Limb(R) for pediatric cerebral palsy: A case report. Brain Dev..

[18] Russell D.J., Rosenbaum P.L., Cadman D.T., Gowland C., Hardy S., Jarvis S. (1989). The gross motor function measure: A means to evaluate the effects of physical therapy. Dev. Med. Child Neurol..

[19] Franjoine M.R., Gunther J.S., Taylor M.J. (2003). Pediatric balance scale: A modified version of the berg balance scale for the school-age child with mild to moderate motor impairment. Pediatr. Phys. Ther..

[20] Maher C.A., Williams M.T., Olds T.S. (2008). The six-minute walk test for children with cerebral palsy. Int. J. Rehabil. Res..

[21] Nsenga Leunkeu A., Shephard R.J., Ahmaidi S. (2012). Six-minute walk test in children with cerebral palsy gross motor function classification system levels I and II: Reproducibility, validity, and training effects. Arch. Phys. Med. Rehabil..

[22] Thompson P., Beath T., Bell J., Jacobson G., Phair T., Salbach N.M., Wright F.V. (2008). Test-retest reliability of the 10-metre fast walk test and 6-minute walk test in ambulatory school-aged children with cerebral palsy. Dev. Med. Child. Neurol..

[23] Unnithan V.B., Clifford C., Bar-Or O. (1998). Evaluation by exercise testing of the child with cerebral palsy. Sports Med..

[24] Corry I.S., Duffy C.M., Cosgrave A.P., Graham H.K. (1996). Measurement of oxygen consumption in disabled children by the Cosmed K2 portable telemetry system. Dev. Med. Child. Neurol..

[25] Maltais D.B., Robitaille N.M., Dumas F., Boucher N., Richards C.L. (2012). Measuring steady-state oxygen uptake during the 6-min walk test in adults with cerebral palsy: Feasibility and construct validity. Int. J. Rehabil. Res..

[26] Lusardi M.M., Jorge M., Nielsen C.C. (2013). Orthotics and Prosthetics in Rehabilitation-E-Book.

[27] Oeffinger D., Bagley A., Rogers S., Gorton G., Kryscio R., Abel M., Damiano D., Barnes D., Tylkowski C. (2008). Outcome tools used for ambulatory children with cerebral palsy: Responsiveness and minimum clinically important differences. Dev. Med. Child Neurol..

[28] Tudor-Locke C., Schuna J.M., Han H., Aguiar E.J., Larrivee S., Hsia D.S., Ducharme S.W., Barreira T.V., Johnson W.D. (2018). Cadence (steps/min) and intensity during ambulation in 6–20 year olds: The CADENCE-kids study. Int. J. Behav. Nutr. Phys. Act..

[29] Wessels M., Lucas C., Eriks I., de Groot S. (2010). Body weight-supported gait training for restoration of walking in people with an incomplete spinal cord injury: A systematic review. J. Rehabil. Med..

[30] Borggraefe I., Schaefer J.S., Klaiber M., Dabrowski E., Ammann-Reiffer C., Knecht B., Berweck S., Heinen F., Meyer-Heim A. (2010). Robotic-assisted treadmill therapy improves walking and standing performance in children and adolescents with cerebral palsy. Eur. J. Paediatr. Neurol..

[31] Willoughby K.L., Dodd K.J., Shields N., Foley S. (2010). Efficacy of partial body weight-supported treadmill training compared with overground walking practice for children with cerebral palsy: A randomized controlled trial. Arch. Phys. Med. Rehabil..

[32] Ueno T., Watanabe H., Kawamoto H., Shimizu Y., Endo A., Shimizu T., Ishikawa K., Kadone H., Ohto T., Kamada H. (2019). Feasibility and safety of Robot Suit HAL treatment for adolescents and adults with cerebral palsy. J. Clin. Neurosci..

[33] Hanna S.E., Bartlett D.J., Rivard L.M., Russell D.J. (2008). Reference curves for the Gross Motor Function Measure: Percentiles for clinical description and tracking over time among children with cerebral palsy. Phys. Ther..

[34] Beckung E., Carlsson G., Carlsdotter S., Uvebrant P. (2007). The natural history of gross motor development in children with cerebral palsy aged 1 to 15 years. Dev. Med. Child Neurol..

[35] Lotze M., Braun C., Birbaumer N., Anders S., Cohen L.G. (2003). Motor learning elicited by voluntary drive. Brain.

[36] Johnston T.E., Moore S.E., Quinn L.T., Smith B.T. (2004). Energy cost of walking in children with cerebral palsy: Relation to the Gross Motor Function Classification System. Dev. Med. Child Neurol..

[37] Cimolin V., Galli M., Piccinini L., Berti M., Crivellini M., Turconi A.C. (2007). Quantitative analysis of gait pattern and energy consumption in children with cerebral palsy. J. Appl. Biomater. Biomech..

[38] Bell K.L., Davies P.S. (2010). Energy expenditure and physical activity of ambulatory children with cerebral palsy and of typically developing children. Am. J. Clin. Nutr..

[39] Batista K.G., de Oliveira Lopes P., Serradilha S.M., de Souza G.A.F., Bella G.P., de Souza R.C.T. (2010). Benefícios do condicionamento cardiorrespiratório em crianças ou adolescentes com paralisia cerebral. Fisioterapia em Movimento.

[40] Maltais D.B., Wiart L., Fowler E., Verschuren O., Damiano D.L. (2014). Health-related physical fitness for children with cerebral palsy. J. Child Neurol..

[41] ATS Committee on Proficiency Standards for Clinical Pulmonary Function Laboratories (2002). ATS statement: Guidelines for the six-minute walk test. Am. J. Respir. Crit. Care Med..

[42] Grecco L.A., Zanon N., Sampaio L.M., Oliveira C.S. (2013). A comparison of treadmill training and overground walking in ambulant children with cerebral palsy: Randomized controlled clinical trial. Clin. Rehabil..

[43] Bayon C., Martin-Lorenzo T., Moral-Saiz B., Ramirez O., Perez-Somarriba A., Lerma-Lara S., Martinez I., Rocon E. (2018). A robot-based gait training therapy for pediatric population with cerebral palsy: Goal setting, proposal and preliminary clinical implementation. J. Neuroeng. Rehabil..

